# Polypharmacy Prevalence and Perceptions of Deprescribing in Elderly Patients Among Community Primary Care Providers in Mid-Michigan Practices

**DOI:** 10.7759/cureus.39399

**Published:** 2023-05-23

**Authors:** Jad Zreik, Mark Kato, Beth Bailey, Wendy S Biggs, Ghassan Hamadeh

**Affiliations:** 1 Internal Medicine, Central Michigan University College of Medicine, Saginaw, USA; 2 Public Health, Michigan Health Improvement Alliance, Midland, USA; 3 Health Services Research, Central Michigan University College of Medicine, Saginaw, USA; 4 Family Medicine, Central Michigan University College of Medicine, Mount Pleasant, USA; 5 Family Medicine, Central Michigan University College of Medicine, Saginaw, USA

**Keywords:** mid-michigan, elderly, primary care, deprescribing, polypharmacy

## Abstract

Introduction: Polypharmacy is common among the elderly and can predispose them to increased morbidity and higher healthcare expenditures. Deprescribing is an important aspect of preventative medicine to minimize polypharmacy-related adverse effects. Mid-Michigan has historically been considered a medically underserved area. We sought to describe polypharmacy prevalence and primary care provider (PCP) perceptions of deprescribing in the elderly at community practices in the region.

Methods: Medicare Part D claims data from 2018 to 2020 were queried to calculate the prevalence of polypharmacy, which is defined as Medicare beneficiaries who were concurrently prescribed at least five medications. PCPs from four community practices in adjacent counties in mid-Michigan, including two high- and two low-prescribing practices, were surveyed to assess their perceptions of deprescribing.

Results: The prevalence of polypharmacy in two adjacent mid-Michigan counties was 44.0% and 42.5%, which was similar to Michigan’s overall prevalence of 40.7% (p = 0.720 and 0.844, respectively). Additionally, 27 survey responses were received from mid-Michigan PCPs (response rate, 30.7%). Most respondents expressed confidence in deprescribing in the elderly from a clinical standpoint (66.7%). Barriers to deprescribing included patient/family concerns (70.4%) and lack of time during office visits (37.0%). Facilitators to deprescribing included patient readiness (18.5%), collaboration with case managers/pharmacists (18.5%), and up-to-date medication lists (18.5%). An exploratory comparison of perceptions at high- and low-prescribing practices showed no significant differences.

Conclusion: These findings demonstrate a high prevalence of polypharmacy in mid-Michigan and suggest that PCPs in the region are generally supportive of deprescribing. Potential targets to improve deprescribing in patients with polypharmacy include addressing visit length, patient/family concerns, increasing interdisciplinary collaboration, and medication reconciliation support.

## Introduction

Polypharmacy, typically defined as the daily use of five or more unique medications, is common among the elderly, given their high prevalence of multiple chronic comorbidities [1,2]. A nationally representative sample of older community-dwelling adults in the United States in 2011 found that 35.8% of them concurrently took at least five prescription medications, whereas 67.1% of them concurrently took at least five prescription medications, non-prescription medications, or supplements of any kind [3]. These rates of prescription drug use and polypharmacy have been continuously and significantly increasing at the national level [3,4]. Patients experiencing polypharmacy may also have a high prevalence of unnecessary drug use. For example, 58.6% of older veteran outpatients experiencing polypharmacy at a single medical center were taking at least one unnecessary medication [5].

Older adults are particularly vulnerable to adverse effects from inappropriate polypharmacy due to their physiologic profiles [6,7]. Age-related changes in metabolism may alter the pharmacokinetics and pharmacodynamics of many drugs, including decreased renal clearance, slower hepatic metabolism, and changes in volume of distribution. These changes may increase the risk of drug-drug interactions, adverse drug reactions over time, and medication-related hospitalizations [6-8]. Cognitive impairment, declining functional status, and greater fall risk are associated with polypharmacy in geriatric patients [9-11]. Polypharmacy has also been associated with higher total healthcare expenditures due to the cost of greater prescription drug use as well as increased outpatient visits and hospitalizations [12,13].

Many of the risks related to polypharmacy may be minimized by deprescribing, which is defined as the process of dose reduction or complete withdrawal of a medication that is no longer indicated. Deprescribing by primary care providers (PCPs) at routine wellness visits is seen as a crucial aspect of preventative medicine, especially for elderly patients, as their health status evolves over time. Previous reports have indicated that older adults are highly amenable to deprescribing if recommended by their physician [14,15]. Physicians, however, often cite cultural, patient-related, and institution-specific barriers to deprescribing in the elderly [16,17]. Current literature evaluating facilitators and barriers to deprescribing is predominantly limited to international settings that may not reflect community medical settings in the United States due to differences in healthcare policies and practices [18,19]. Significant regional variations in the prevalence of polypharmacy may also occur within the country [20,21].

Identifying community-specific considerations toward deprescribing at the physician level is vital for guiding policies and quality improvement initiatives moving forward. Mid-Michigan has historically been considered a medically underserved area in the state where barriers to primary care visits may limit regular medication reviews and deprescribing [22]. Nonetheless, polypharmacy and deprescribing practices in the region, as well as other community settings, have not been well characterized in the medical literature. The aim of this study is to describe the prevalence of polypharmacy and assess PCP perceptions of deprescribing among providers in mid-Michigan community practices.

## Materials and methods

Data source & study participants

This study queried Medicare Part D claims data from the Centers for Medicare and Medicaid Services (CMS) for the rate of polypharmacy for all Medicare beneficiaries at the county level in the state of Michigan from 2018 to 2020. The data were mapped to Provider Enrollment, Chain, and Ownership System (PECOS) ID codes to identify the rates of polypharmacy at the practice level. Polypharmacy was defined as Medicare beneficiaries who were concurrently prescribed at least five distinct medications.

PCPs at four community practices in two adjacent counties in mid-Michigan were surveyed for their perceptions on deprescribing. The counties were chosen as examples of regional counties in the bottom and top quartiles of health status in Michigan, according to the University of Wisconsin Population Health Institute, in order to represent varied levels of health status [23]. They also border each other and have an overlap on where people work, live, and seek their medical care. A high-prescribing and low-prescribing practice was included from each county to account for potential variations in primary care practice. High- and low-prescribing practices were defined as practices in the top and bottom quartiles of polypharmacy rates, respectively.

Survey design

An electronic survey was created using Qualtrics (Qualtrics, Provo, UT). The survey was distributed to all PCPs at each of the identified practices using a standardized email invitation with an embedded survey link. A unique survey link was used for each practice. A reminder email was sent two weeks following the initial distribution of the survey to encourage non-responders to complete the survey. Surveys were completed and submitted anonymously from April 5, 2022, to May 4, 2022. Respondent demographics were collected and included age, sex, medical specialty, and number of years in practice. The survey contained nine items to assess PCP-reported attitudes toward deprescribing in the elderly on a 5-point Likert scale from “strongly disagree” to “strongly agree,” as previously described and validated by Djatche et al. [24]. Free text prompts on barriers and facilitators to deprescribing were also included.

Data analysis

The prevalence of polypharmacy was calculated from the Medicare Part D claims data as the percentage of Medicare beneficiaries concurrently prescribed at least five medications in the state of Michigan and each county in the state. Chi-squared goodness of fit was used to compare the prevalence of polypharmacy between regions. From the survey results, continuous variables were reported as means with standard deviations (SDs) or medians and interquartile ranges for non-normal distributions. Categorical variables were reported as counts with proportions. An exploratory analysis comparing perceptions toward deprescribing at high- and low-prescribing practices was also performed, given the low sample size. Demographic and survey data were compared using the Wilcoxon test for continuous variables and Fisher’s exact test for categorical variables. Statistical analysis was conducted using the R statistical programming language (version 4.0.5) including the “usmap” and “ggplot2” packages [25]. The p-values <0.05 were considered statistically significant.

## Results

Claims data results

**Figure 1:**
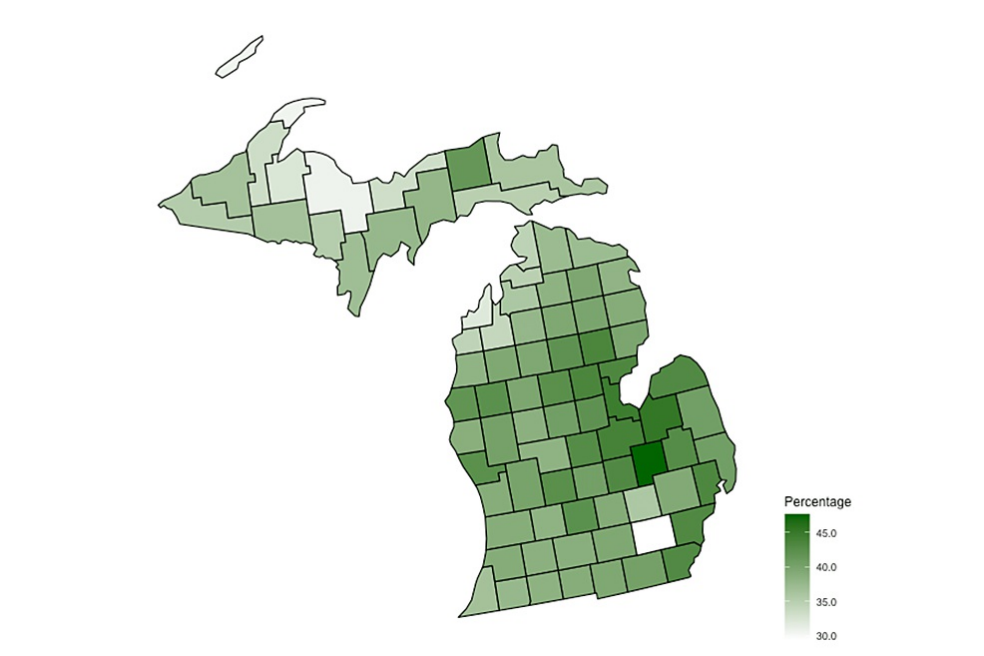
Prevalence of polypharmacy at the county level in Michigan from 2018 to 2020

The prevalence of polypharmacy in Medicare recipients in Michigan was found to be 40.7%, which is similar to the national average of 38.5% (p = 0.805). The prevalence of polypharmacy in the two surveyed, adjacent mid-Michigan counties was 44% and 42.5%, which were similar to the overall statewide prevalence (p = 0.720 and 0.844, respectively). The prevalence between the two adjacent counties was also similar (p = 0.872). Additionally, there was substantial variability in polypharmacy rates across the state (Figure 1).

Survey results

Surveys were distributed to a total of 88 PCPs at the selected primary care practices. Overall, 27 responses were received (response rate, 30.7%). The average age of the respondents was 39.8 years (SD, 9.5) with most being female (n = 18, 66.7%). The majority were also family medicine physicians (n = 25, 92.6%) and had ≤10 years of experience in practice (n = 17, 63.0%). In addition, 17 respondents (63.0%) were from a high-prescribing practice, while 10 respondents (37.0%) were from a low-prescribing practice (Table 1).

**Table 1:** Respondent demographics

	Total (n = 27)
Age, average (SD)	39.8 (9.5)
Sex, n (%)	
Male	9 (33.3%)
Female	18 (66.7%)
Medical specialty, n (%)	
Family medicine	25 (92.6%)
Internal medicine	1 (3.7%)
Nurse practitioner	1 (3.7%)
Years in practice, n (%)	
≤10	17 (63.0%)
11-20	4 (14.8%)
20+	6 (22.2%)
Practice prevalence of polypharmacy, n (%)	
High-prescribing	17 (63.0%)
Low-prescribing	10 (37.0%)

Two-thirds of all respondents (66.7%) expressed confidence in deprescribing in the elderly from a clinical standpoint. Most respondents favored deprescribing when the life expectancy of elderly patients no longer justified the potential benefits of certain medications (n = 23, 85.2%) and in patients with poor life expectancy despite guidelines recommending certain medications (n = 20, 74.1%). Furthermore, 40.7% (n = 11) agreed that a lack of robust evidence in favor of continuation or cessation prevented them from deprescribing. Most respondents also expressed confidence in deprescribing medications initially prescribed by another physician (n = 16, 59.3%). PCPs generally disagreed when asked if they had problems in deprescribing when their patients believe that the continuation of medications is needed (n = 19, 70.4%) and with not considering deprescribing due to fear of adverse drug withdrawal effects (n = 20, 74.1%). Most agreed that they have no difficulty in motivating their elderly patients to engage in deprescribing (n = 15, 55.6%). The majority of respondents, however, agreed that they do not have the necessary time to engage in deprescribing despite its importance (n = 20, 74.1%) (Table 2).

**Table 2:** Provider attitudes toward deprescribing in the elderly from survey prompts

Prompt	Strongly disagree (1)	Somewhat disagree (2)	Neither agree nor disagree (3)	Somewhat agree (4)	Strongly agree (5)	Median (Q1, Q3)
From a clinical standpoint, I feel confident with deprescribing in my elderly patients	0 (0%)	9 (33.3%)	0 (0%)	14 (51.9%)	4 (14.8%)	4 (2, 4)
When the life expectancy of my elderly patients no longer justifies potential benefits, I am in favor of deprescribing preventative medications	1 (3.7%)	3 (11.1%)	0 (0%)	8 (29.6%)	15 (55.6%)	5 (4, 5)
In elderly patients with poor life expectancy, it would be appropriate to consider deprescribing therapeutic medications even though they are recommended by guidelines	1 (3.7%)	4 (14.8%)	2 (7.4%)	10 (37.0%)	10 (37.0%)	4 (3.5, 5)
In elderly patients, lack of robust evidence in favor of continuation or cessation of preventative medications prevents me from deprescribing	0 (0%)	6 (22.2%)	10 (37.0%)	10 (37.0%)	1 (3.7%)	3 (2.5, 4)
In my elderly patients, I have no hesitation in deprescribing medications initially prescribed by another physician	1 (3.7%)	8 (29.6%)	2 (7.4%)	11 (40.7%)	5 (18.5%)	4 (2, 4)
I do not have the necessary time to spend with my elderly patients and/or caregivers to effectively undertake the process of deprescribing medications even though I consider it important	0 (0%)	4 (14.8%)	3 (11.1%)	14 (51.9%)	6 (22.2%)	4 (3.5, 4)
I have no problems in deprescribing medications even if my elderly patients and/or caregivers believe continuation is needed	2 (7.4%)	17 (63%)	1 (3.7%)	7 (25.9%)	0 (0%)	2 (2, 3.5)
Although in certain situations I may consider appropriately deprescribing medications to my elderly patients, but I do not consider it for fear of adverse drug withdrawal effects	4 (14.8%)	16 (59.3%)	4 (14.8%)	3 (11.1%)	0 (0%)	2 (2, 2.5)
I have no difficulty to motivate my elderly patients and/or caregivers in order to engage them in the process of deprescribing medications	4 (14.8%)	4 (14.8%)	4 (14.8%)	10 (37%)	5 (18.5%)	4 (2, 4)

Provider perceptions regarding barriers and facilitators toward deprescribing were also assessed using open-ended questions in the survey. In 70% of comments, respondents mentioned patient and family concerns about discontinuing medications as a barrier to deprescribing (n = 19, 70.4%). Other barriers noted in respondents’ comments were lack of time during the office visit (n = 10, 37.0%), medications prescribed by multiple specialists (n = 3, 11.1%), accuracy of medication lists (n = 2, 7.4%), and deprescribing guideline-recommended medications (n = 2, 7.4%). Facilitating factors mentioned to assist deprescribing were the patient’s willingness to deprescribe (n = 5, 18.5%), collaboration with case managers and pharmacists (n = 5, 18.5%), up-to-date medication lists (n = 5, 18.5%), strong patient relationships (n = 4, 14.8%), and evidence-based recommendations to facilitate deprescribing (n = 4, 14.8%) (Tables 3, 4).

**Table 3:** Provider perceptions on barriers toward deprescribing in the elderly from free-response survey prompt

Barriers toward deprescribing	n (%)
Patient/family concerns	19 (70.4%)
Lack of time during office visits	10 (37.0%)
Medications prescribed by multiple specialists	3 (11.1%)
Accuracy of patient medication list	2 (7.4%)
Deprescribing guideline-recommended medications	2 (7.4%)

 

**Table 4:** Provider perceptions on facilitators toward deprescribing in the elderly from free-response survey prompt

Facilitators toward deprescribing	n (%)
Patient willingness toward deprescribing	5 (18.5%)
Partnership with staff (i.e., case managers and clinical pharmacists)	5 (18.5%)
Current and up-to-date medication list from medication reconciliation surveys and “brown bag visits”	5 (18.5%)
Trust, rapport, and overall relationship with patient	4 (14.8%)
Evidence-based recommendations and tools for deprescribing	4 (14.8%)
Time for medication review and patient education	3 (11.1%)
None available	2 (11.1%)

High- vs low-prescribing

A comparative analysis of survey responses from high- vs low-prescribing practices was limited to an exploratory analysis due to the small sample size. Overall, the characteristics of respondents from each practice type were similar (all p > 0.05) (Table 5). Survey responses also demonstrated no significant differences in perceptions toward deprescribing (all p > 0.05) (Table 6).

**Table 5:** Demographics of survey respondents at high- vs low-prescribing survey practices

	High-prescribing (n = 17)	Low-prescribing (n = 10)	p-value
Age, average (SD)	40.9 (10.1)	37.9 (8.6)	0.481
Sex, n (%)			0.406
Male	7 (41.2%)	2 (20.0%)	
Female	10 (58.8%)	8 (80.0%)	
Medical specialty, n (%)			0.613
Family medicine	16 (94.1%)	9 (90.0%)	
Internal medicine	1 (5.8%)	0 (0%)	
Nurse practitioner	0 (0%)	1 (10.0%)	
Years in practice, n (%)			0.506
≤10	10 (58.8%)	8 (80.0%)	
11-20	3 (17.6%)	0 (0%)	
20+	4 (23.5%)	2 (20.0%)	

 

**Table 6:** Provider attitudes toward deprescribing at high- vs low-prescribing practices *Median (Q1, Q3) calculated based on survey responses from the 5-point Likert scale

Prompt	High-prescribing (n = 17)*	Low-prescribing (n = 10)*	p-value
From a clinical standpoint, I feel confident with deprescribing in my elderly patients	4 (2, 4)	4 (2.5, 4)	1.00
When the life expectancy of my elderly patients no longer justifies potential benefits, I am in favor of deprescribing preventative medications	5 (4, 5)	5 (5, 5)	0.881
In elderly patients with poor life expectancy, it would be appropriate to consider deprescribing therapeutic medications even though they are recommended by guidelines	4 (3, 5)	4 (4, 4.8)	0.700
In elderly patients, lack of robust evidence in favor of continuation or cessation of preventative medications prevents me from deprescribing	3 (3, 4)	3 (3, 3.8)	0.802
In my elderly patients, I have no hesitation in deprescribing medications initially prescribed by another physician	4 (2, 4)	4 (2.3, 4)	0.956
I do not have the necessary time to spend with my elderly patients and/or caregivers to effectively undertake the process of deprescribing medications even though I consider it important	4 (3, 5)	4 (4, 4)	0.627
I have no problems in deprescribing medications even if my elderly patients and/or caregivers believe continuation is needed	2 (2, 3)	2 (2, 3.5)	1.00
Although in certain situations I may consider appropriately deprescribing medications to my elderly patients, but I do not consider it for fear of adverse drug withdrawal effects	2 (2, 3)	2 (2, 2)	1.00
I have no difficulty to motivate my elderly patients and/or caregivers in order to engage them in the process of deprescribing medications	4 (2, 4)	3 (2.3, 4)	0.417

## Discussion

In the current study, we sought to evaluate the prevalence of polypharmacy in mid-Michigan as well as the perceptions of PCPs in two counties in mid-Michigan toward deprescribing in the elderly. The prevalence of polypharmacy in adjacent counties in mid-Michigan appeared high at 44.0% and 42.5%, which were similar to the statewide prevalence of 40.7%. A survey of PCPs in the region found that they were generally confident in deprescribing unnecessary medications, but considerations such as patient concerns about deprescribing, limited time during visits to engage patients, and perceived lack of evidence assisting physicians to deprescribe a single medication served as barriers. Utilizing staff and other health professionals and having accurate medication lists were cited as facilitators of deprescribing.

The high prevalence of polypharmacy in both counties is likely due to a multitude of factors. Michigan has a shortage of PCPs, which decreases access to regular visits in which deprescribing could be emphasized. PCPs may have less time available per visit and may prioritize current patient concerns and comorbidities over deprescribing discussions. Outside of the metro areas in southeastern and western Michigan, Michigan is rural. Rural areas tend to have a limited number of resources, making it less likely that case managers and pharmacists are available to augment the deprescribing process [26]. The general health status in mid-Michigan, where patients have a high prevalence of comorbidities compared to the rest of the state and country, may also lend itself to a higher rate of polypharmacy [23].

The findings from our survey focus on community practices in mid-Michigan and add to the growing body of evidence on improving deprescribing practices. At the patient level, factors such as the appropriateness and process of medication cessation were identified as key factors in deprescribing cardiovascular medications [27]. A cross-sectional analysis of questionnaires by healthcare providers in Singapore found that knowledge, available time during the office visit, and communication between PCPs and specialists contributed to the process of deprescribing [28], which is similar to our results. Furthermore, patient and family concerns about deprescribing were identified as both a top facilitator and barrier to deprescribing in our analysis. Incorporating more targeted patient education as a mechanism to address patient and family concerns has also been studied to further facilitate the process in the clinic [29].

There are some limitations to this study. The physician demographics may have impacted the results. Two-thirds of respondents self-identified as female, whereas the national average of physicians reported by the Association of American Medical Colleges (AAMC) in 2021 is 58% male and 42% female [30]. Also, the majority of the physicians surveyed had practiced for less than 10 years. The attitudes toward deprescribing between early-, mid-, and late-career physicians may differ. Practices with higher rates of polypharmacy may have had a patient population with a greater number of comorbidities, thereby requiring additional medications for management. Therefore, adjacent counties in the top and bottom quartiles of overall state health status were selected as a means to represent a spectrum of baseline patient health. Despite the contrast in overall health status between the selected counties, analysis of the Medicare Part D claims data demonstrated that county-level polypharmacy rates were similar in the two adjacent counties (44.0% vs 42.5%). Additionally, the survey was distributed to practices in mid-Michigan, so the generalizability outside of the region may be limited. The small number of clinics surveyed also limits generalizability. Analysis of the facilitators and barriers to deprescribing were also limited to the survey questions and free-response answers. Not all physicians included free-response comments, which may bias our findings. There may be additional facilitators or barriers that were not included in the survey questions or the free text comments. Finally, the comparison of perceptions toward deprescribing at high- vs low-prescribing practices was limited to an exploratory analysis due to low sample size.

## Conclusions

In a regional analysis, these findings demonstrate a high prevalence of polypharmacy in mid-Michigan and suggest that PCPs in the region are generally supportive of deprescribing. Potential targets to improve deprescribing in patients with polypharmacy include addressing visit length, patient/family concerns, increasing interdisciplinary collaboration, and medication reconciliation support. These findings may help guide further research and policies to improve the practice of deprescribing and lower the rate of polypharmacy in the elderly at community primary care practices.
